# Using Data Mining to Assist in Predicting Reproductive Outcomes Following Varicocele Embolization

**DOI:** 10.3390/jcm10163503

**Published:** 2021-08-09

**Authors:** Ana Paula Sousa, Judith Santos-Pereira, Maria José Freire, Belmiro Parada, Teresa Almeida-Santos, Jorge Bernardino, João Ramalho-Santos

**Affiliations:** 1 Biology of Reproduction & Stem Cell Group, CNC-Center for Neuroscience and Cell Biology, CIBB, University of Coimbra, Azinhaga de Santa Comba, Celas, 3004-504 Coimbra, Portugal; anapauladesousa.apms@gmail.com (A.P.S.); anateresasantos.tas@gmail.com (T.A.-S.); 2 Reproductive Medicine Unit, Centro Hospitalar e Universitário de Coimbra, Pr. Prof Mota Pinto, 3004-561 Coimbra, Portugal; mjfreire.urologia@gmail.com (M.J.F.); parada.belmiro@gmail.com (B.P.); 3 Polytechnic of Coimbra, ISEC-Instituto Superior de Engenharia de Coimbra, R. Pedro Nunes, 3030-199 Coimbra, Portugal; santosj@hotmail.ca (J.S.-P.); jorge@isec.pt (J.B.); 4 Urology Unit, Centro Hospitalar e Universitário de Coimbra, Pr. Prof Mota Pinto, 3004-561 Coimbra, Portugal; 5 Faculty of Medicine, University of Coimbra, Azinhaga de Santa Comba, Celas, 3000-548 Coimbra, Portugal; 6 Centre for Informatics and Systems, University of Coimbra, Polo II, Pinhal de Marrocos, 3030-290 Coimbra, Portugal; 7 Department of Life Sciences, University of Coimbra, Calçada Martim de Freitas, 3000-456 Coimbra, Portugal

**Keywords:** varicocele, embolization, data mining, sperm parameters

## Abstract

We carried out a retrospective analysis of infertile couple data using several methodologies and data analysis techniques, including the application of a novel data mining approach for analyzing varicocele treatment outcomes. The aim of this work was to characterize embolized varicocele patients by ascertaining the improvement of some of their clinical features, predicting the success of treatment via pregnancy outcomes, and identifying data patterns that can contribute to both ongoing varicocele research and the more effective management of patients treated for varicocele. We retrospectively surveyed the data of 293 consenting couples undergoing infertility treatment with male varicocele embolization over a 10-year period, and sperm samples were collected before and at 3, 6, and 12 months after varicocele embolization treatment and analyzed with World Health Organization parameters—varicocele severity grades were assessed with medical assessment and scrotal ultrasound, patient personal information (e.g., age, lifestyle, and embolization complications) was collected with clinical inquiries, and varicocele embolization success was measured through pregnancy outcomes. Varicocele embolization significantly improved sperm concentration, motility, and morphology mean values, as well as sperm chromatin integrity. Following this study, we can predict that a male patient without a high varicocele severity grade (with grade I or II) has a 70.83% chance of conceiving after embolization treatment if his partners’ age is between 24 and 33 with an accuracy of 70.59%. Furthermore, male patients successful in achieving pregnancy following embolization are mostly characterized by having a normal sperm progressive motility before treatment, a normal sperm concentration after treatment, a moderate to low varicocele severity grade, and not working in a putatively hazardous environment.

## 1. Introduction

Varicocele is characterized by the dilation of the veins of the spermatic cord [1] in one (unilateral) or both (bilateral) testis. Studies estimate that varicocele is present in more than 35% of infertile couples [2]. The McGraw-Hill Concise Dictionary of Modern Medicine goes even further by stating that varicocele is present in 40% of males treated for infertility. Given that infertility affects an estimated 15% of couples globally [3] and male infertility factors are responsible for 50% of infertility causes [2], the importance of assessing the data of patients with such a prevalent condition is clear.

It has been suggested that varicocele triggers several inflammatory pathways that negatively affect spermatogenesis [4], ultimately reflected in alterations in sperm quality [5]. The European Association of Urology (EUA) recommends that, in order to improve fertility rates, varicocele should be treated in infertile men with a clinical varicocele, abnormal semen parameters, and otherwise unexplained infertility in couples where the female partner has a good ovarian reserve [6]. For this purpose, several approaches have been suggested. One of the suggested treatments is the use of antioxidant supplementation to neutralize the oxidative stress caused by the activated inflammatory pathways. However, in addition to the existence of contradictory literature, the authors of a recent was concluded that this approach does not seem to improve either seminal parameters or pregnancy rates [7]. More effective results seem to be obtained when varicocele is treated by surgical varicocelectomy [6]. However, this is an invasive technique. Minimally invasive techniques for varicocele correction include the radiological embolization technique that introduces substances (such as coils, sclerosants, or glue) into the circulation to devitalize the enlarged veins [8]. It has been shown that embolization successfully corrects varicocele, whether with coils or glue, in an average of 92% of cases [9]. Furthermore, varicocele correction has an important impact on the treatment of infertility [10] since it has been found to lead to clinical reproductive outcome improvement of parameters, such as sperm quality and pregnancy rate, when comparing patients undergoing varicocele correction with those with the untreated condition [2,11,12].

However, despite the existing evidence, the link between varicocele and male infertility remains controversial, as it is possible to find this condition in men with normal fertility. The same is true when the benefit of varicocele correction is analyzed [13]. Due to the heterogeneity of varicocele patterns and responses to its treatment, other advanced data analysis techniques, such as the use of data mining, may improve existing knowledge linked to varicocele and identify new insights regarding this infertility treatment. To the best of our knowledge, only traditional statistics has been used when assessing the efficacy of varicocele treatment in the context of human infertility.

Data mining is the process of discovering data patterns [14] where standard statistical exploratory data analysis procedures (traditional statistics) cannot offer useful insights [15]. In this field, traditional statistics are usually viewed as the primary data analysis technique, with data mining as a putatively relevant secondary technique due to its characteristics [16]. While the groundwork of both techniques is mathematics, data mining extends its range over topics such as machine learning, database systems, and visualization, which brings important gains over traditional statistics techniques [17]. The main advantages of data mining over traditional statistics methods are its capability to analyze different types of data (e.g., numbers, names, and severity degrees) and its ability to perform inductive analysis. This latter is fundamental in cases where researchers are trying to understand, for instance, the consequences of a treatment that are not fully known since many potential variables can hinder the formulation of a hypothesis to be validated or rejected [16].

In this context, through a retrospective analysis, the aim of this work was to characterize embolized varicocele patients by ascertaining the improvement of some of their clinical features, predicting the success of treatment via pregnancy outcomes, and identifying data patterns that can contribute to both ongoing varicocele research and the more effective management of patients treated for varicocele. To tackle these goals, we used statistical techniques (traditional approach) and then applied the most commonly used data mining techniques in healthcare. Though these data mining techniques have proven applicability in healthcare [17,18,19,20], they are rarely applied together in the medical treatment domain, as shown in the meta-analysis carried out by Tomar et al. [19]. To the best of our knowledge, such an advanced data analysis technique has never been applied to embolized varicocele patients.

## 2. Materials and Methods

All patients signed informed consent forms, and all human material was used in accordance with the appropriate ethical and internal review board (IRB) guidelines provided by the Centro Hospitalar e Universitário de Coimbra (ID number CHUC-115-12).

### 2.1. Description of Population

Data analysis was retrospectively carried out on a dataset of 293 infertile couples (i.e., couples that were unable to get pregnant after 1 year of regular intercourse) where male partners had undergone varicocele embolization between January 2007 and April 2016 in a Portuguese public hospital—the Reproductive Medicine Unit of the Centro Hospitalar e Universitário de Coimbra (CHUC)—with the aim of improving their chances of conceiving. Patients that did not perform a sperm analysis before the varicocele embolization and patients without partner were excluded from the analysis. Because a large percentage of the population achieved pregnancy after varicocele correction, not all female partners were thoroughly characterized.

The classification of varicocele was assessed by medical examination by one of the two physicians involved and/or by Doppler ultrasound, according to the European Association of Urology guidelines [6]:

- Subclinical: not palpable or visible at rest or during Valsalva maneuver, but can be shown by Doppler ultrasound.
- Grade 1: palpable during Valsalva maneuver.
- Grade 2: palpable at rest.
- Grade 3: visible and palpable at rest

### 2.2. Sperm Preparation and Analyses

Patients were evaluated through a routine semen analysis before varicocele treatment, as well as 3, 6, and 12 months after varicocele embolization. Semen samples were obtained by masturbation after 3–5 days of sexual abstinence and collected in the center, and routine seminal analysis was performed by one of the six available technicians according to the World Health Organization Guidelines [21]. Among other parameters (volume, pH, presence of leucocytes, and sperm agglutination), sperm parameters such as concentration (million sperm/mL), progressive motility (percentage of progressively motile sperm), and morphology (percentage of morphologically normal sperm) were assessed. Sperm chromatin status was also evaluated using the Diff-Quik like stain set (Dade Behring Inc., Newark, NJ, USA), as previously described [22], where the percentage of sperm with damaged chromatin (i.e., either decondensed or presenting fragmented DNA) was determined.

### 2.3. Follow-Up

Further data were obtained using follow-up inquiries and medical record assessments. The occurrence of complications after varicocele embolization and global patient satisfaction with the technique were evaluated. Complications were graded according to Clavien–Dindo classification [23]. The achievement of clinical pregnancy, both spontaneously or using assisted reproductive techniques (ART), was also assessed using follow-up inquiries and medical record assessments.

### 2.4. Statistics

Using the embolized varicocele patient sample data, we first applied statistical techniques to understand the collected data and ascertain the evolution of sperm parameter mean values, as well as chromatin integrity after embolization.

All patient features were described and explored statistically as suggested by Han et al. [14] to successfully prepare the data for the mining process. In fact, these authors stated that one should analyze the central tendency of data (i.e., compute the mean, median, and mode of each attribute), as well as its dispersion (i.e., compute the minimum value (Min), the value of the first quartile (Q_1_), the value of the third quartile (Q_3_), the maximum value (Max), and the standard deviation (SD) of each patient feature). Hence, this study computed all these statistical measures and complemented the assessment by generating graphs such as time series, box plots, and histograms with the RapidMiner data mining platform and Excel software to better understand the collected attributes.

The chi-square and the ANOVA tests were used to assess the sperm parameter values for statistical inference, and appropriate post-hoc tests were used to identify which sperm parameter mean values were statistically significantly different. The Kruskal–Wallis and Mann–Whitney statistical tests were used to assess the statistical significance between the clustered patient’s mean feature values. *p* ≤ 0.05 was considered statistically significant.

### 2.5. Data Mining

Following the traditional statistical approach, we then applied the most commonly used data mining techniques in healthcare: classification, using a decision tree algorithm; clustering, using the k-means algorithm; and association, with the FP-growth algorithm. Though these data mining techniques have proven applicability in healthcare [17,18,19,20], they are rarely applied together in the medical treatment domain, as shown in the meta-analysis carried out by Tomar et al. [19].

Data mining was achieved by following the cross-industry standard process for data mining project (CRISP-DM) methodology [24]. This involves a set of six phases that can be recursively executed and are briefly described as covering the following tasks:

- Business understanding: determine the business objectives and data mining goals.
- Data understanding: collect, describe, and explore the data, as well as verify their quality.
- Data preparation: select, clean, construct, integrate, and format the previously understood data.
- Modeling: select data mining techniques, define the training/testing design, build the data mining models, and assess them with performance measures.
- Evaluation: assess data models with respect to business objectives and domain expertise.
- Deployment: disclosure of results.

In the modeling phase, the selected data mining techniques were the most common in the healthcare domain (i.e., classification, clustering, and association), as previously described. Thus, with the use of the RapidMiner data mining platform, we applied the following algorithms: decision tree algorithm for the classification technique, k-means algorithm for the clustering technique, and FP-growth algorithm for the association technique.

## 3. Results

**Figure 1:**
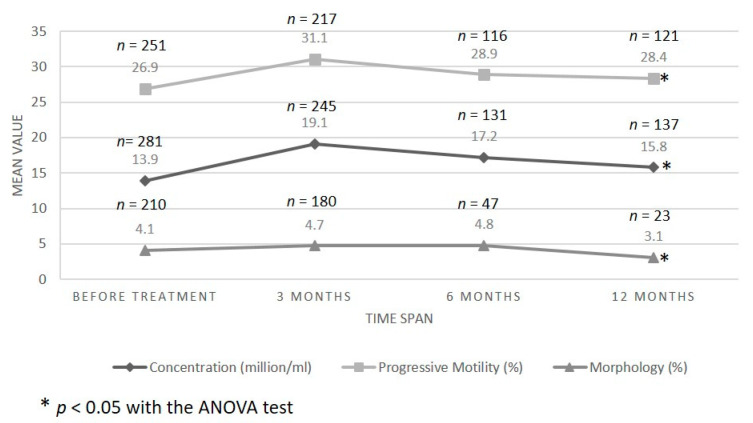
Evolution of sperm parameter mean values according to patient follow-up time. Sperm concentration, progressive motility, and morphology were assessed according to WHO criteria [21].

**Figure 2:**
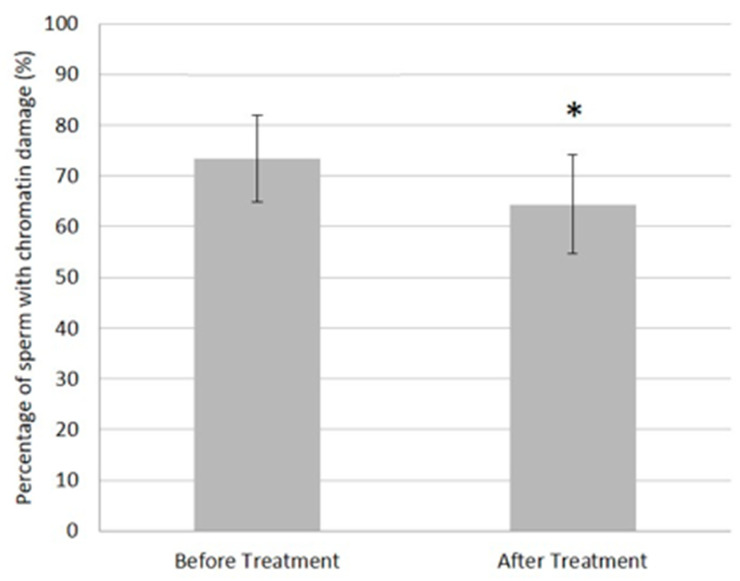
Evolution of sperm chromatin integrity. Sperm chromatin integrity was monitored before and after varicocele embolization, as described in the Methods section. * *p* = 0.043. Sperm chromatin integrity was assessed as previously described [22]. *n* = 86.

Though there was significant inter-patient variability, semen analysis revealed that, before embolization, the population of patients with varicocele had values of sperm concentration (13.9 ± 23.6 million sperm/mL) and progressive motility (26.9 ± 22.5%) below the WHO [21] reference values (Figure 1). Similarly, sperm chromatin integrity was also affected (73.4 ± 8.5% of sperm with damaged chromatin; Figure 2). More importantly, sperm parameter mean values (Figure 1) improved after embolization (*p* = 0.00006, *p* = 0.003, and *p* = 0.05 at 3 months after embolization for sperm concentration, motility, and morphology, respectively). However, sperm concentration was the only parameter that remained significantly improved (ANOVA *p* = 0.017) a year after varicocele correction. Chromatin integrity (Figure 2) also improved after varicocele embolization (*p* = 0.043), but it was still higher (64.4 ± 9.7%) than the reference value for normality (<32%) that was previously described using the same methodology [22].

Out of the 230 couples that we were able to determine a pregnancy outcome, 107 (i.e., 46.52%) got pregnant; of these, 54.21% (58/107) conceived with an ART procedure, 38.31% (41/107) conceived spontaneously. The remaining 7.48% (8/107) conceived both naturally and via ART, considering at least two different pregnancies. Interestingly, almost 80% of the pregnancies were obtained in the first year after embolization. During the time of the study, 78.50% (84/107) of couples had a single pregnancy, 17.75% (19/107) had two pregnancies, and 3.74% (4/107) had three pregnancies after the embolization treatment.

Importantly, patients that were able to conceive had a statistically significantly better sperm progressive motility before the embolization, with a mean difference of 6.9 (ANOVA *p* = 0.018); better sperm morphology 3 months after the embolization, with a mean difference of 1.6 (ANOVA *p* = 0.004); and increased sperm concentration 6 months after the embolization, with a mean difference of 8.2 (ANOVA *p* = 0.015) (Table 1).

The patient features that were significantly related with pregnancy outcome were: male partner age (ANOVA *p* = 0.018), varicocele severity grade (chi-square *p* = 0.049), sperm concentration at 6 months following embolization (ANOVA *p* = 0.015), sperm progressive motility before treatment (ANOVA *p* = 0.018), sperm morphology at 3 months (ANOVA *p* = 0.004), sperm concentration normality at 3 months following embolization (chi-square *p* = 0.017), sperm progressive motility normality before treatment (chi-square *p* = 0.027), sperm progressive motility normality at 3 months following embolization (chi-square *p* = 0.022), semen quality classification before treatment (chi-square *p* = 0.017), semen quality classification at 3 months following embolization (chi-square *p* = 0.018), and a potentially hazardous occupation when considering possible exposure to known toxicants (chi-square *p* = 0.023).

**Figure 3:**
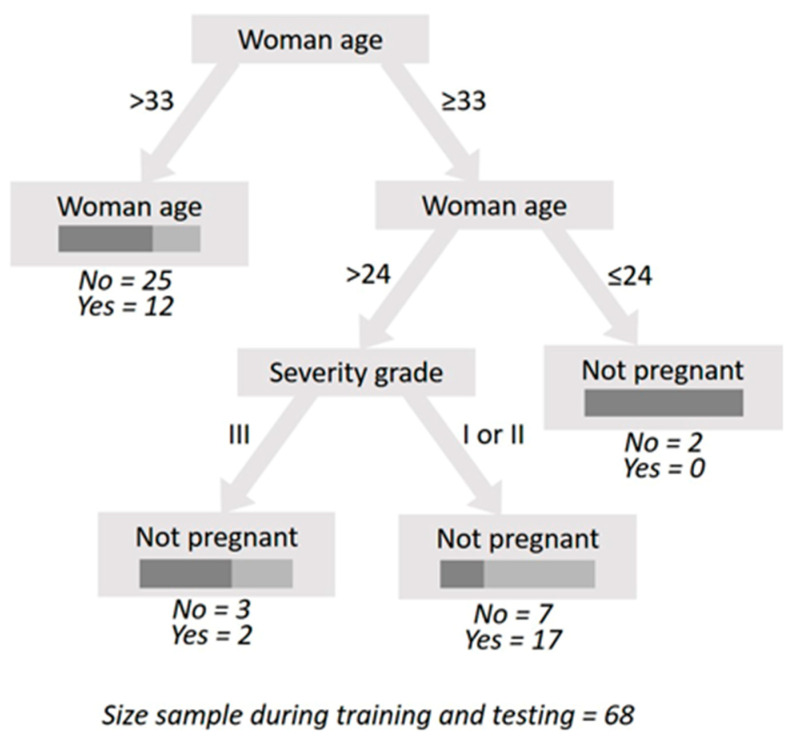
Decision tree for the prediction of the varicocele embolization success. The test design entailed the sub-sampling of 85 patients: 48 patients for training; 20 patients for testing, and 17 patients for validating the decision tree. This computed decision tree was obtained with the following parameter values after exhaustive training/testing: sampling type during training/testing = linear; decision tree splitting criterion = accuracy; decision tree pruning = true; decision tree minimal size for split = 4; decision tree minimal gain = 0.1; decision tree minimal leaf size = 2; and decision tree maximal depth = 20. During its training/testing, the decision tree computed the following performance measures: accuracy = 80.00%; precision = 85.71%; recall = 66.67%; F-measure = 75.00%; and AUC = 0.717. During its validation, the performance measures were: accuracy = 70.59%; precision = 66.67%; recall = 75.00%; F-measure = 70.59%; and AUC = 0.750 with the confusion matrix shown in Table 2.

Varicocele embolization success, defined as whether the man´s partner got pregnant, was able to be predicted with an accuracy of 70.59% by stating that a male patient without a high varicocele severity grade (with grade I or II) has a 70.83% chance (i.e., (17/(7 + 17) = 70.83%) of conceiving after the embolization treatment if his partner is between the ages of 24 and 33 years old inclusively (Figure 3). The results in Figure 3 suggest that male patients with a female partner aged 24 or younger are still not able to conceive. This suggests that the cause for unsuccessful pregnancy following embolization in these cases might involve female infertility, since this decision branch does not entail a male patient feature, as is the case when female partner age is above 33.

**Figure 4:**
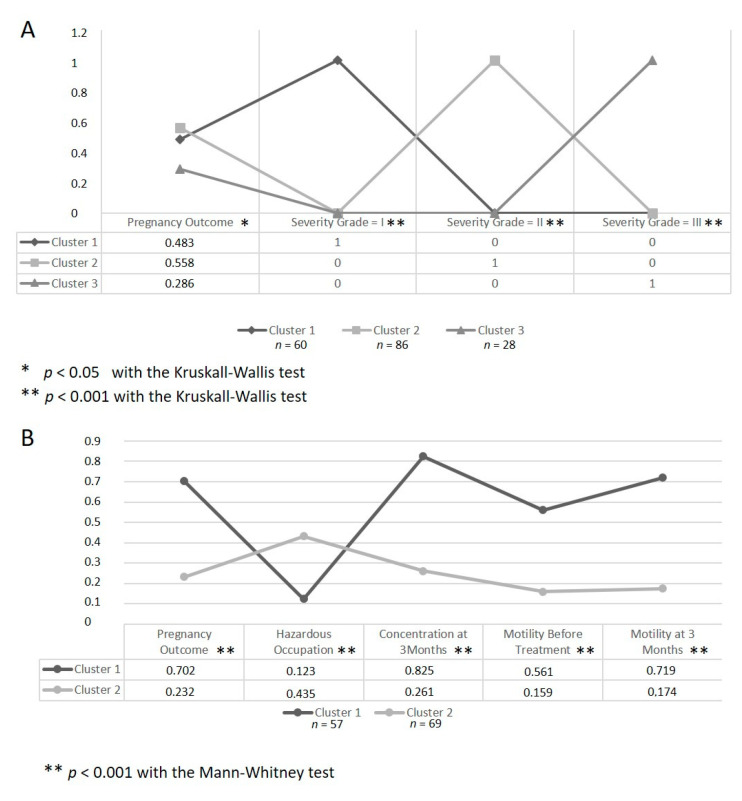
Data patterns related with the pregnancy outcome: (**A**) implication of the varicocele severity grade; (**B**) implication of a putative hazardous occupation and the normality of sperm parameter values.

By applying the k-means algorithm to clusters of patients with similar and well-defined characteristics (namely the 174 patients where both pregnancy outcomes and severity grade were available (Figure 4A) and the 126 patients where simultaneous information on pregnancy outcomes, putative hazardous occupation, and the normality of the statistically significant sperm parameter values was available (Figure 4B)), we were able to identify these data patterns for successfully embolized patients (Figure 4). Please note that since the k-means algorithm groups patients that are similar to each other in clusters and all patient features were normalized/mapped, the mean value of each cluster per each feature can be interpreted as the percentage of patients with a positive event. Indeed, in this analysis, the value of 1 was attributed to positive events and the value of 0 was attributed to negative events; hence, a positive pregnancy outcome, a specific severity grade, a patient working in a putative hazardous environment, and a patient having a sperm parameter value above the World Health Organization threshold all imply a value of 1 [21]. Specifically, regarding data pattern identification, for the 174 patients shown in Figure 4A, the algorithm selected three clusters showing that patients with a mild to medium varicocele severity grade are significantly (Kruskal–Wallis *p* = 0.041) more prone to conceive than the ones with a high severity grade (i.e., severity grade I = 48.3% pregnancy and severity grade II = 55.8% pregnancy vs severity grade III = a clearly lower chance of 28.6% in terms of chances of conceiving). Furthermore, the data of 126 patients in Figure 4B show that patients with most successful embolization rarely work in putative hazardous occupations (12.3% vs. 43.5%) and are more likely to have a normal sperm concentration (82.5% vs. 26.1%) and normal progressive motility (71.9% vs. 17.4%) at 3 months after varicocele correction with a statistically significant difference (Mann–Whitney *p* = 0.0009).

Interestingly, although putative contact with hazardous environments seems to have an effect, other male patient lifestyle habits, such as drinking or smoking, or a previous disease or surgery did not seem to influence the success of treatment.

Furthermore, all couples that were able to spontaneously conceive had at least one of the sperm parameter categorized as normal or a previously diagnosed moderate varicocele condition at 3 months following embolization. In fact, the FP-growth algorithm computed the following rules:

1. Severity grade = II → spontaneous pregnancy = yes (*p* ≤ 0.10 *n* = 107)
  support = 0.252, confidence = 0.562, lift = 1.228, conviction = 1.239, chi-square = 3.82
2. Sperm concentration at 3 months following embolization ≥ 15 → spontaneous pregnancy = yes (*p* > 0.10 *n* = 107)
  support = 0.262, confidence = 0.519, lift = 1.132, conviction = 1.126, chi-square = 1.61
3. Progressive motility at 3 months ≥ 32 → spontaneous pregnancy = yes (*p* ≤ 0.05 *n* = 107)
  support = 0.262, confidence = 0.560, lift = 1.223, conviction = 1.232, chi-square = 3.95
4. Morphology at 3 months ≥ 4 → spontaneous pregnancy = yes (*p* ≤ 0.10 *n* = 107)
  support = 0.224, confidence = 0.517, lift = 1.248, conviction = 1.265, chi-square = 3.58

However, based on the obtained chi-square values, unlike other sperm parameters, a normal sperm concentration at 3 months following embolization was not significantly related to spontaneous conception despite the lift measure indicating that both events are codependent (i.e., lift > 1).

Finally, regarding the feedback from the embolized patients, from the 292 responses obtained after varicocele embolization treatment, 270 patients (92.47%) had not experienced any complications. The complications reported in the 22 remaining patients were all classified as Grade I according to Clavien–Dindo classification [23], where 13 out of the 22 patients reported to have experienced some pain. Moreover, most patients (93.29%) reported that they would redo the procedure.

## 4. Discussion

Varicocele has been pointed out as one of the main causes of male infertility. Though the exact mechanism that leads to sperm impairments is not totally known, classical sperm parameters are impaired and sperm chromatin integrity is compromised, and it was recently demonstrated that varicocele leads to oxidative stress-induced sperm DNA damage [25]. However, in an era that has seen a preponderance of ICSI (intracytoplasmic sperm injection) in terms of ART procedures, the benefit of varicocele correction has been questioned. In this study, we have seen that varicocele embolization improves all sperm parameter mean values, namely (and in the long run) sperm concentration. Accordingly, a recent analysis [9] showed that all its reviewed studies reported improved sperm parameters after varicocele embolization. Similarly, the benefit of varicocele repair in sperm DNA damage has also been reported [26].

This improvement has been suggested as important towards achieving pregnancy. After treatment, almost 47% of the patients obtained a pregnancy, either spontaneously or through ART, and 80% of them did so in the first year after embolization. Similarly, others have reported that varicocele repair increased pregnancy and live birth rates [12]. Reproductive outcomes are associated with not only sperm parameters found before treatment but also improvements obtained after embolization, as we demonstrated using data mining techniques. Furthermore, other parameters, such as the age of the female partner, varicocele grade, and a possible hazardous occupation (revealed by decision tree and/or the cluster analysis) seem to also have important roles. Though several studies have analyzed the association between varicocele/varicocele treatment and several relevant parameters, including sperm parameters [6], but their putative predictor values have never been assessed.

However, despite having achieved interesting conclusions in our opinion, this study had some clear limitations. The number of patients that ended up being selected by the data mining algorithm was relatively small. However, after analyzing the literature only 30 clinical studies on varicocele embolization were found (reviewed in [9]). Reviewing the published information our dataset is actually larger than other related works in this field, which have analyzed, on average, 117 patients (±102 patients). In this study, because both spontaneous pregnancy and pregnancy after ART were included, we decided not to exclude any female fertility factors, as ART overcomes almost all these factors. However, we are aware that this may have hindered the analysis of the results. Furthermore, as this was a retrospective study, no control group (e.g., placebo, antioxidant treatment, or varicocelectomy) was included. Therefore, a larger prospective study should be conducted in order to confirm our findings.

## 5. Conclusions

The combined use of statistical and data mining techniques can contribute towards finding patterns associated with varicocele, since several parameters mat be influencing the success of its treatment and thus muddle analyses. In fact, although several studies on this condition have been carried out, none of them have assessed a varicocele database with a non-hypothetic-deductive technique such as data mining in order to identify novel patient features that can positively influence the success of varicocele correction and, hence, contribute to the improvement of male infertility treatments. To our knowledge, this is the first such study.

We were able to identify interesting data patterns with different algorithms (k-means and FP-growth), which suggests that the success of varicocele embolization, evaluated through the achievement of a pregnancy/live birth, is positively influenced by the presence of a normal sperm progressive motility before treatment, a normal sperm concentration after treatment, a moderate to low varicocele severity grade, a young male patient age, and a male patient occupation that does not involve contact with putatively toxic environments or products.

Considering the results, and although ICSI is always a therapeutic possibility in cases of severe male infertility, it is important to treat the male factor in order to increase the chances of conceiving, either spontaneously or by ART, especially when treatment is possible with a simple, minimally invasive, and patient-friendly approach.

## Figures and Tables

**Table 1:** Pregnancy outcome vs sperm parameter mean values through patient’s follow up times.

Sperm Parameter		Pregnancy Outcome	*n*	Mean	Mean Difference	SD
Concentration	Before treatment	Yes	107	14.5	−0.4	21.6
		No	119	14.9		27.2
	3 months	Yes	94	22.9	4.8	24.2
		No	107	18.1		27.5
	6 months **	Yes	50	22.9	8.2	29.5
		No	65	14.7		20.6
	12 months	Yes	60	18.8	4.3	19.5
		No	69	14.5		18.2
Progressive Motility	Before treatment **	Yes	102	29.9	6.9	23.3
		No	107	23.0		20.8
	3 months	Yes	92	33.2	3.3	21.6
		No	92	29.9		21.1
	6 months	Yes	49	33.5	6.2	25.1
		No	57	27.3		25.6
	12 months	Yes	58	30.8	4.1	23.6
		No	58	26.7		22.8
Morphology	Before treatment	Yes	89	4.0	0.4	5.0
		No	87	3.6		3.5
	3 months ***	Yes	78	5.5	1.6	5.0
		No	74	3.9		3.4
	6 months	Yes	22	5.0	0.5	3.2
		No	19	4.5		5.2
	12 months ***	Yes	10	4.0	1.1	3.3
		No	12	2.9		1.4

** ANOVA *p* < 0.05; *** ANOVA *p* < 0.01.

**Table 2:** Decision tree’s confusion matrix.

Actual	Predicted	
	Yes	No
Yes	7	1
No	3	6

The target variable (pregnancy) has two values: positive (yes) or negative (no). The columns represent the actual values of the target variable. The rows represent the predicted values of the target variable.

## Data Availability

All available data are included in the manuscript.
